# Supplementary material for: Trait heritability in major transitions
Source: BMC Biol. 2018 Dec 13;16:145. doi: 10.1186/s12915-018-0612-6 (PMC6293664; doi:10.1186/s12915-018-0612-6)
Supplement: Supplementary file 1 — Ratios of sums of squares as approximations for ratios of variances. Justifies the use of sums of squares as approximations for variances and gives a numerical example showing that inaccuracies introduced by these approximations will normally be negligible. (DOCX 20 kb) [file 12915_2018_612_MOESM1_ESM.docx]

**Ratios of sums of squares as approximations for ratios of variances**

For the heritability of particle-level traits, we are interested in the ratio

$\frac{s_{A}^{2}}{s_{A}^{2}+s_{B/A}^{2}+s_{C/B}^{2}}$ **(1)**

which is broad-sense heritability. Start with the variance formulae for nested ANOVA (balanced design):

$s_{A}^{2}=\frac{MSA-MS\left( B/A \right)}{cb}$ **(2)**

$s_{B}^{2}=\frac{MS\left( B/A \right)-MS\left( C/B \right)}{c}$ **(3)**

$s_{C}^{2}=MS\left( C/B \right)$ **(4)**

Substitute in formulae for mean squares:

$s_{A}^{2}=\frac{\frac{SSA}{a-1}-\frac{SS\left( B/A \right)}{a\left( b-1 \right)}}{cb}$ **(5)**

$s_{B}^{2}=\frac{\frac{SS\left( B/A \right)}{a\left( b-1 \right)}-\frac{SS\left( C/B \right)}{ab\left( c-1 \right)}}{c}$ **(6)**

$s_{C}^{2}=\frac{SS\left( C/B \right)}{ab\left( c-1 \right)}$ **(7)**

and the ratio is

$\frac{s_{A}^{2}}{s_{A}^{2}+s_{B/A}^{2}+s_{C/B}^{2}}=\frac{\frac{\frac{SSA}{a-1}-\frac{SSB}{a\left( b-1 \right)}}{cb}}{\frac{\frac{SSA}{a-1}-\frac{SS\left( B/A \right)}{a\left( b-1 \right)}}{cb}+\frac{\frac{SS\left( B/A \right)}{a\left( b-1 \right)}-\frac{SS\left( C/B \right)}{ab\left( n-1 \right)}}{c}+\frac{SS\left( C/B \right)}{ab\left( c-1 \right)}}$ **(8)**

Multiply by *cb*/*cb*:

$\frac{s_{A}^{2}}{s_{A}^{2}+s_{B/A}^{2}+s_{C/B}^{2}}=\frac{\frac{SSA}{a-1}-\frac{SS\left( B/A \right)}{a\left( b-1 \right)}}{\frac{SSA}{a-1}-\frac{SS\left( B/A \right)}{a\left( b-1 \right)}+b\frac{SS\left( B/A \right)}{a\left( b-1 \right)}-b\frac{SS\left( C/B \right)}{ab\left( c-1 \right)}+cb\frac{SS\left( C/B \right)}{ab\left( c-1 \right)}}$ **(9)**

Simplify the SSE terms in the denominator:

$\frac{s_{A}^{2}}{s_{A}^{2}+s_{B/A}^{2}+s_{C/B}^{2}}=\frac{\frac{SSA}{a-1}-\frac{SS\left( B/A \right)}{a\left( b-1 \right)}}{\frac{SSA}{a-1}-\frac{SS\left( B/A \right)}{a\left( b-1 \right)}+b\frac{SS\left( B/A \right)}{a\left( b-1 \right)}-\frac{SS\left( C/B \right)}{a\left( c-1 \right)}+c\frac{SS\left( C/B \right)}{a\left( c-1 \right)}}$ **(10)**

Group like terms in the denominator:

$\frac{s_{A}^{2}}{s_{A}^{2}+s_{B/A}^{2}+s_{C/B}^{2}}=\frac{\frac{SSA}{a-1}-\frac{SS\left( B/A \right)}{a\left( b-1 \right)}}{\frac{SSA}{a-1}+\left( b-1 \right)\frac{SS\left( B/A \right)}{a\left( b-1 \right)}+\left( c-1 \right)\frac{SS\left( C/B \right)}{a\left( c-1 \right)}}$ **(11)**

Simplify SS(B/A) and SS(C/B) terms in the denominator:

$\frac{s_{A}^{2}}{s_{A}^{2}+s_{B/A}^{2}+s_{C/B}^{2}}=\frac{\frac{SSA}{a-1}-\frac{SS\left( B/A \right)}{a\left( b-1 \right)}}{\frac{SSA}{a-1}+\frac{SS\left( B/A \right)}{a}+\frac{SS\left( C/B \right)}{a}}$ **(12)**

As *a* and *b* grow large, *a*/(*a*-1) approaches unity, and the second term of the numerator becomes negligible, so $\frac{s_{A}^{2}}{s_{A}^{2}+s_{B/A}^{2}+s_{C/B}^{2}}$ approaches $\frac{SSA}{SSA+SS\left( B/A \right)+SS\left( C/B \right)}$.

For the heritability of collective-level traits, we are interested in the ratio

$\frac{s_{\alpha}^{2}}{s_{\alpha}^{2}+s_{\beta/\alpha}^{2}}$ **(13)**

which is broad-sense heritability. Start with the variance formulae for one-way ANOVA:

$s_{\alpha}^{2}=\frac{MS\alpha-MS\left( \beta/\alpha\right)}{b}$ **(14)**

$s_{\beta/\alpha}^{2}=MS\left( \beta/\alpha\right)$ **(15)**

Substitute in formulae for mean squares:

$s_{\alpha}^{2}=\frac{\frac{SS\alpha}{a-1}-\frac{SS\left( \beta/\alpha\right)}{a\left( b-1 \right)}}{b}$ **(16)**

$s_{\beta/\alpha}^{2}=\frac{SS\left( \beta/\alpha\right)}{a\left( b-1 \right)}$ **(17)**

and the ratio is

$\frac{s_{\alpha}^{2}}{s_{\alpha}^{2}+s_{\beta/\alpha}^{2}}=\frac{\frac{\frac{SS\alpha}{a-1}-\frac{SS\left( \beta/\alpha\right)}{a\left( b-1 \right)}}{b}}{\frac{\frac{SS\alpha}{a-1}-\frac{SS\left( \beta/\alpha\right)}{a\left( b-1 \right)}}{b}+\frac{SS\left( \beta/\alpha\right)}{a\left( b-1 \right)}}$ **(18)**

Multiply by *b*/*b*:

$\frac{s_{\alpha}^{2}}{s_{\alpha}^{2}+s_{\beta/\alpha}^{2}}=\frac{\frac{SS\alpha}{a-1}-\frac{SS\left( \beta/\alpha\right)}{a\left( b-1 \right)}}{\frac{SS\alpha}{a-1}-\frac{SS\left( \beta/\alpha\right)}{a\left( b-1 \right)}+b\frac{SS\left( \beta/\alpha\right)}{a\left( b-1 \right)}}$ **(19)**

Group like terms in the denominator:

$\frac{s_{\alpha}^{2}}{s_{\alpha}^{2}+s_{\beta/\alpha}^{2}}=\frac{\frac{SS\alpha}{a-1}-\frac{SS\left( \beta/\alpha\right)}{a\left( b-1 \right)}}{\frac{SS\alpha}{a-1}+\left( b-1 \right)\frac{SS\left( \beta/\alpha\right)}{a\left( b-1 \right)}}$ **(20)**

Simplify the SS(β/α) term in the denominator:

$\frac{s_{\alpha}^{2}}{s_{\alpha}^{2}+s_{\beta/\alpha}^{2}}=\frac{\frac{SS\alpha}{a-1}-\frac{SS\left( \beta/\alpha\right)}{a\left( b-1 \right)}}{\frac{SS\alpha}{a-1}+\frac{SS\left( \beta/\alpha\right)}{a}}$ **(21)**

As *a* and *b* grow large, *a*/(*a*-1) approaches unity, and the second term of the numerator becomes negligible, so $\frac{s_{\alpha}^{2}}{s_{\alpha}^{2}+s_{\beta/\alpha}^{2}}$ approaches $\frac{SS\alpha}{SS\alpha+SS\left( \beta/\alpha\right)}$.

How large is large? Consider the following numerical example: for the approximation in (12), the nested model, let SSA = 30, SS(B/A) = 20, and SS(C/B) = 10. If *a* = *b* = 5, the true heritability (ratio of variances) is 0.481, while that from sums of squares is 0.500, an approximation that would make any quantitative geneticist happy. At *a* = *b* = 10, the approximation differs from the true value by 0.009; at *a* = *b* = 20, by 0.004. These outcomes will of course depend on the particular values; we provide these examples only to give a flavor of the magnitude of the difference. All three examples represent small and genetically depauperate populations, for example 20 clones each containing 20 collectives. In most real biological populations the difference between the approximation and the true heritability will be negligible.
