# Supplementary material for: Trait heritability in major transitions
Source: BMC Biol. 2018 Dec 13;16:145. doi: 10.1186/s12915-018-0612-6 (PMC6293664; doi:10.1186/s12915-018-0612-6)
Supplement: Supplementary file 6 — Detailed description of the derivation of Eq. 15. (DOCX 25 kb) [file 12915_2018_612_MOESM6_ESM.docx]

**Swimming Velocity:**

We model the upward swimming speed (*V_up_*) of a spherical algal colony of radius *R* following Solari *et al.* [1]. Table 1 shows the parameters and variables used in this model.

Table 1. Model parameters and variables.

| Symbol | Definition |
| --- | --- |
| *N* | Number of cells per colony |
| *R* | Radius of a colony |
| *r* | Average radius of cells in a colony |
| *M* | Mass of a colony |
| *a* | Vertical acceleration of a colony |
| *V_up_* | Upward swimming velocity of a colony |
| *ΔM* | Difference between colony mass and mass of the water it displaces |
| *Δρ_c_* | Density difference between cells and water (0.047g/cm^3^) |
| *f* | Average effective flagellar force per cell (2.4 × 10^-7^ g⋅cm/s^2^) |
| *Δρ* | Density difference between a colony and water |
| *η_w_* | Water viscosity (0.01 g/cm⋅s) |
| *A* | Intercellular area |

There are there are three forces acting on the collective. First, the Stokes drag force for a sphere moving in a fluid with viscosity *η_w_:*

$F_{s}=6\pi\eta_{w}RV_{up}$ (**1**)

This force is always opposite to the moving direction of the sphere. The second force is the buoyancy force, which is equal to *gΔM* where *g* is gravitational acceleration and *ΔM* is the mass difference between the collective and the water it displaces:

$\Delta M=\frac{4}{3} \pi r^{3}\Delta\rho N$ (**2**)

where *Δρ_c_* is the density difference between cells and water, *r* is the average cell radius and *N* is the total number of cells in the collective. Volvocine algae are denser than water, so buoyancy is always negative. The last force is the cells’ swimming force (*Nf*) where *f* is the average effective swimming force per cell. Therefore the equation of motion of a collective would be:

$6\pi\eta_{w}RV_{up} + g\Delta M - Nf = Ma$ (**3**)

where *a* is the acceleration of the collective in the vertical direction. Using simple geometric model developed by Solari et al. 2006 for the radius of the collective as a function of cells within that collective we have:

$R\approx\frac{1}{2} \sqrt{\left( r^{2}+A \right)N}$ (**4**)

where *A* is the intercellular area (which we assume is zero). When the collective is swimming with constant speed, its acceleration is zero and the upward swimming force is equal to the sum of drag and buoyancy forces:

$Nf =6\pi\eta_{w}R V_{up} + g\Delta M$ (**5**)

Solving equation 5 for *V_up_* and using equations 2 and 4:

$V_{up}=\left( \frac{fN^{0.5}}{3\pi\eta_{w}} \right)r^{-1} - \left( \frac{g\Delta\rho_{c}\left( \frac{4}{3} \right)N^{2}}{3\eta_{w}} \right)r^{2}$ (**6**)

Using the numerical values of parameters in Solari et al. [1], *V_up_* for a collective as a function of radius of cells in a 32-celled colony is:

$V_{up}$*=*(0.02/*π*)r^-1^-(400/3)r^2^ (**7**)

**References:**

1. Solari CA, Kessler JO, Michod RE. A hydrodynamics approach to the evolution of multicellularity: flagellar motility and germ-soma differentiation in volvocalean green algae. Am Nat. 167:537–54. Available from: http://www.jstor.org/stable/info/10.1086/501031
